# Effects of different doses of omega-3 polyunsaturated fatty acids on gut microbiota and immunity

**DOI:** 10.29219/fnr.v65.6263

**Published:** 2021-07-08

**Authors:** Xueliang Zhu, Zhichao Bi, Chen Yang, Yanhui Guo, Jieli Yuan, Longjie Li, Yanjie Guo

**Affiliations:** 1 State Key Laboratory of Advanced Technology for Materials Synthesis and Processing, Wuhan University of Technology, Wuhan, China; 2 Department of Microecology, School of Basic Medical Science, Dalian Medical University, Dalian, China; 3 Oil Crops Research Institute, Chinese Academy of Agricultural Sciences, Wuhan, China; 4 Key Laboratory of Oilseeds Processing, Ministry of Agriculture and Rural Affairs, Wuhan, China; 5 Department of Radiation Oncology, the First affiliated hospital of Dalian Medical University, Dalian, Liaoning, China

**Keywords:** omega-3 PUFAs, microbiota, immunity, dose, ceftriaxone sodium

## Abstract

**Background:**

Omega-3 polyunsaturated fatty acids (PUFAs) play beneficial roles in metabolism and health. Little is known about the effects of different doses of omega-3 PUFAs on gut microbiota.

**Objective:**

In this study, we focus on the effects of different doses of omega-3 PUFAs on gut microbiota and immunity.

**Design:**

BALB/c mice was first treated with ceftriaxone sodium for 7 days, and then they received saline or different doses of omega-3 PUFAs (30, 60 and 90 mg omega-3 PUFAs) via daily gavage for 21 days. Alterations of cecum microbiota; the tight junction proteins, zonula occludens 3 (ZO3) and occludin, in the ileal wall; serum lipopolysaccharide (LPS); Interleukin-10 (IL-10), interleukin-1β (IL-1β), and Tumour Necrosis Factor α (TNF-α) ; mucus SIgA levels were measured.

**Results:**

Compared with the ceftriaxone sodium administration group, significant increases in bacterial richness and diversity were observed in the 60- and 90-mg omega-3 PUFA groups, while only a slight increase was observed in the 30-mg omega-3 PUFA group. A higher percentage of several genera, including *Lactobacillus*, *Helicobacter,* and *Ruminococcus*, and a lower percentage of *Bacteroides*, *Clostridium,* and *Prevotella* were observed in the 60- and 90-mg omega-3 PUFA groups when compared with those in the 30-mg group. The expression of ZO3 and occludin proteins increased in 60- and 90-mg omega-3 PUFA groups compared with the natural recovery group. The mucus SIgA and serum IL-10 levels were increased, and serum levels of LPS, IL-1β, and TNF-α were decreased in the 60- and 90-mg omega-3 PUFA groups when compared with those in the ceftriaxone sodium-treated group.

**Conclusion:**

Different doses of omega-3 PUFAs have different therapeutic effects on the intestinal microbiota. The 60- and 90-mg omega-3 PUFA supplementation had better recovery effects on the gut microbiota and immunity than those of the 30 mg omega-3 PUFAs supplementation.

## Popular scientific summary

- Omega-3 PUFA supplementation was beneficial to restore the gut microbiota and regulate the immunity in ceftriaxone sodium-treated mice.
- Different doses of omega-3 PUFAs could affect different microbiota and intestinal wall permeability, thereby having an impact on the host immune response.
- The 60- and 90 mg omega-3 PUFAs have better recovery effects than the 30-mg group, which indicated that only sufficient doses can play an effective role.

Long-chain omega-3 polyunsaturated fatty acids (PUFAs), such as docosahexaenoic acid (DHA C22:6) and eicosapentaenoic acid (EPA C20:5), are important constituents of the phospholipids of cell membranes for maintaining membrane fluidity and flexibility. Omega-3 PUFAs can be synthesized from the essential fatty acid, alpha-linolenic acid (ALA, C18:3), or taken from the diet. It has been reported that omega-3 PUFAs play beneficial effects on human health, including in neurodevelopment (1), eye function (2), and liver lipid metabolism (3). Omega-3 PUFA supplements are used in the treatment or prevention of diseases, such as depression, cardiovascular diseases (5), and inflammatory bowel disease (IBD) (6). The beneficial effects of omega-3 PUFAs on human health require adequate doses and duration, as a low dose of omega-3 PUFAs (180 mg of EPA and 120 mg of DHA) for 8 weeks does not have significant effects on inflammatory and oxidative stress markers in people with type 2 diabetes (7), while a higher dose of omega-3 PUFAs (1,200 mg DHA + EPA) for 12 weeks could improve insulin sensitivity and reduce the triglyceride levels in individuals with a high risk of type 2 diabetes (8). Moreover, with regard to effects of omega-3 PUFAs on cardiovascular diseases, according to the literature, the dose also affects the therapeutic effect (9, 10). In this context, different doses of omega-3 PUFAs have different effects in treating diseases, and the underlying mechanisms need to be investigated.

Gut microbiota are composed of thousands of microorganisms, which normally play an important physiological role in metabolism and immunity. Change in the intestinal microbial structure, including alterations in microbial diversity and abundance, exerts multifaceted effects on the body’s immune system. Considerable studies have revealed that defects in gut microbiota are related to human disorders, including IBD (11), cardiovascular diseases (12), diabetes (13), and liver disease (14). In view of the relationship between microbiota and health and disease, gut microbiota has been used as a subjective measurement for disease and its treatment (15, 16). The few human and animal studies showed that omega-3 PUFA supplementation has an impact on the composition of the gut microbiota (17, 18). Moreover, the beneficial role of omega-3 PUFA supplementation in behavioral disorders may be through regulation of the composition of intestinal microbiota (19), although the research on this topic is still in its initial stages.

Considering the few insights existing in the literature, in this study, we assessed the effects of different doses of omega-3 PUFAs on the recovery of gut microbiota in ceftriaxone sodium-treated mice. We hope that this study will provide some evidence for future research as to why different doses of omega-3 PUFAs have different therapeutic effects.

## Materials and methods

### Animals and diets

**Fig. 1:**
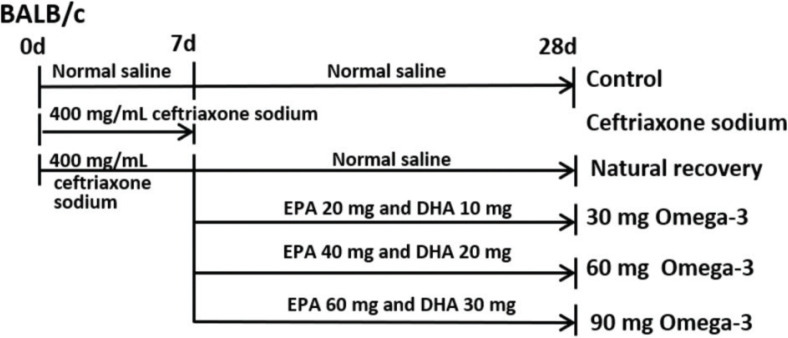
Time lines of the animal experiments. To establish the intestinal microbiota dysbiosis model, BALB/c mice were treated with 0.2 mL ceftriaxone sodium (400 mg/mL) for 7 days. After that, the natural recovery group received a gavage of normal saline, the 30-mg omega-3 PUFAs group (EPA 20 mg and DHA 10 mg), the 60-mg omega-3 PUFAs group (EPA 40 mg and DHA 20 mg), and the 90-mg omega-3 PUFAs group (EPA 60 mg and DHA 30 mg) received a gavage of corresponding dose of omega-3 PUFAs for 21 consecutive days, respectively.

All experimental protocols were approved by the Ethics Committee of Dalian Medical University, China (SCXK-2013-0006) and conformed to the Guide for the Care and Use of Laboratory Animals. Between 6 and 8 weeks of age, specific-pathogen-free (SPF) level inbred male BALB/c mice (18 ± 22 g) were purchased from the Experimental Animal House of Dalian Medical University (*n* = 48). All animals were fed with commercial diet and tap water *ad libitum*. The main ingredients of the commercial diet include protein (195 g/kg), fat (58 g/kg), carbohydrate (610 g/kg), fiber (30 g/kg), calcium (11 g/kg), and total phosphorus (7.5 g/kg); 3.6 kcal g−1. The diet was sterilized by ^60^Co irradiation. The main sources of fat are soybean oil, sunflower oil, and so on (Jiangsu Xietong Pharmaceutical Bioengineering Co. Ltd, Nanjing, China). Mice were randomly divided into six groups of eight mice each: the control mice received a gavage of normal saline; other mice were treated with 0.2 mL ceftriaxone sodium (400 mg/mL) intragastrically twice a day at an interval of 6 h for seven consecutive days. After 7 days of ceftriaxone sodium treatment, the mice in ceftriaxone sodium group were sacrificed; the natural recovery group received a gavage of normal saline, the 30-mg omega-3 PUFA group (EPA 20 mg and DHA 10 mg), the 60-mg omega-3 PUFA group (EPA 40 mg and DHA 20 mg), and the 90-mg omega-3 PUFA group (EPA 60 mg and DHA 30 mg) received a gavage of the corresponding dose of omega-3 PUFAs for 21 consecutive days, respectively. On day 29, all of the mice were sacrificed by decapitation. Various treatments of the animals are shown in Fig. 1.

### Cecum contents microbiota analyses

Different microbes are colonized along the longitudinal axis of the intestine due to their anatomical structure, physical and chemical factors, and nutrients. The small intestine is dominated by fast growing facultative anaerobes, as it has shorter transit times, lower pH, and higher levels of oxygen and antimicrobials agents than the large intestine. However, the large intestine is dominated by anaerobes capable of fermenting complex polysaccharides. Mice have a large cecum between the small and large intestines where plant fibers are slowly digested by the microbiota (20, 21). The great difference in the abundance and diversity of microbiota between the small and large intestines starts from the cecum. In addition, inter-mouse variations of microbiota between the large intestine and fecal samples were much smaller than those between the gastric and small intestine samples (20). Therefore, this research study tested the cecum microbiota of mice to analyze the changes in intestinal microbiota.

After the mice were sacrificed, the cecum samples were collected, and metagenomic DNA was extracted using the QIAamp DNA Stool Mini kit (QIAGEN). The purity and concentration of the DNA were measured using a NanoDrop 2000 spectrophotometer (Thermo Fisher Scientific, USA). The microbial 16S rDNA V3-V4 hypervariable regions were amplified using PCR and used for subsequent analyses. PCR primers for the V3-V4 hyper variable region include 5-ACTCCTACGGGAGGCAGCA-3 and 5-GGACTACHVGGGTWTCTAAT-3. DNA products were analyzed by 2% (wt/vol) agarose gel electrophoresis and purified with the Axygen AP-GX-50 (Axygen, USA). The recovered PCR products were fluorescence quantified using a Quant-iT PicoGreen dsDNA Assay Kit (Invitrogen, USA).

### Bioinformatic analyses

Sequences of the V3-V4 region of 16S rRNA genes were detected using an Illumina MiSeq platform (Personalbio Bioinformatics Technology Co. Ltd, Shanghai, China). Taxon-dependent analysis was carried out using the Greengenes database aided by the Ribosomal Database Project and SILVA database. The operational taxonomic units (OTUs) were counted for each sample to express the richness of bacterial species, with an identity cutoff of 97%. The OTU abundance of each sample was generated at the genus level.

Alpha diversity was assessed to express the richness and evenness of the community using the Simpson, Chao1, ACE and Shannon indices. Beta diversity was analyzed by principal coordinate analysis (PCoA) with weighted or unweighted UniFrac analysis in R software. The linear discriminant analysis effect size (LEfSe) was used in combination with the Kruskal–Wallis and Wilcoxon rank sum test to analyze features with significantly different abundances between assigned taxa. The Linear Discriminant Analysis (LDA) score of > 4.0 was shown as a significantly abundant group in the indicated group.

### Western blot

The total protein was extracted from the ileal tissue of mice using RIPA Lysis buffer (Seven Biotech; China, Beijing) according to the manufacturer’s instructions. The concentrations of the protein samples were determined using the BCA protein assay kit (Beyotime, Shang hai, China). Equal protein was separated on 10% sodium dodecylsulfate polyacrylamide gel electrophoresis (SDS-PAGE) and transferred to a nitrocellulose membrane (Millipore, Billerica, MA). The membranes were incubated with primary antibodies: ZO3 (Abcam, UK; 1:1,000), occludin antibody (Abcam, UK; 1:1,000), and β-actin (Beyotime; China; 1:1,000) at a room temperature for 4 h, followed by incubation with the secondary Horseradish Peroxidase (HRP)-labeled antibody (Beyotime; China; 1:2,000). The optical density of specific bands was detected using an electrochemiluminescence detection system. The relative expression of protein level was quantified by densitometry using the Image J software (NIH, Bethesda, MD).

### Plasma analyses

Levels of serum LPS (Cusabio, Wuhan, China), IL-1β, IL-10, TNF-α, and mucus SIgA were assessed using ELISA assay kits according to the manufacturer’s instructions (Elabscience, Wuhan, China). Sensitivities of assays were estimated to be 0.039 ng/mL, 4.69 pg/mL, 9.38 pg/mL, 18.75 pg/mL and 0.094 ng/mL for LPS, IL-1β, IL-10, TNF-α, and SIgA, respectively. An intestinal mucus sample was collected for SIgA analysis according to the previous study (22).

### Statistical analysis

Statistical analyses were performed using SPSS version 20.0.0 (SPSS Inc., Chicago, IL, USA). Data are represented as mean ± SEM. The statistical significance between groups was compared using one-way analysis of variance (ANOVA) followed by correction of the p values with Dunnett’s post hoc multiple comparisons. The value of *P*< 0.05 was considered to be statistically significant.

## Results

### Overall structural changes in cecal microbiota composition

**Fig. 2:**
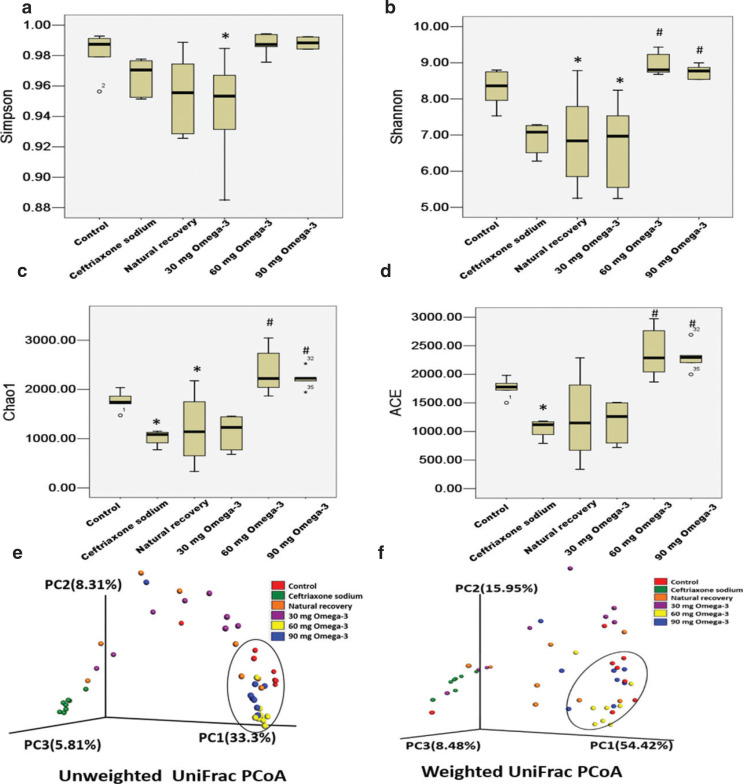
Changes in mice cecal microbiota diversity. Alpha diversity indexes including Simpson (a), Shannon (b), Chao1 (c), and ACE (d) were analyzed. Beta diversity was analyzed by principal coordinate analysis (PCoA) with weighted (e) and unweighted UniFrac (f). Data are presented as mean ± SEM, *P < 0.05 versus control, ^#^*P* < 0.05 versus ceftriaxone sodium group.

Cecal microbiota diversity and richness were assessed using alpha-diversity. The Chao 1 index and ACE index reflect the richness of the community. The higher the Chao1 or ACE index, the higher the richness of the community. The Shannon index and Simpson index reflect the heterogeneity in the community. The higher the Shannon index or Simpson index, the higher the community diversity. After 7 days of administering ceftriaxone sodium, the cecal microbiota richness decreased significantly in the ceftriaxone sodium-treated group when compared with those in the control group. After 21 days of natural recovery or omega-3 PUFA treatment, there were no significant changes in the α-diversity indexes observed in the natural recovery group and the 30-mg omega-3 PUFA group. However, in the 60-mg omega-3 PUFA group and the 90-mg omega-3 PUFA group, the average ACE, Chao, and Shannon estimators increased significantly, which is even slightly higher than that of the control group (Fig. 2a–d).

β-Diversity analysis was carried out to investigate the similarity of community structure among different samples. The abundance matrix obtained from cecal samples was subjected to UniFrac PCoA. As shown in Fig. 2e and f, the 60-mg omega-3 PUFA group and the 90-mg omega-3 PUFA group had the highest similarity with the normal control group.

### Taxonomy-based comparisons at the phylum and genus levels

**Fig. 3:**
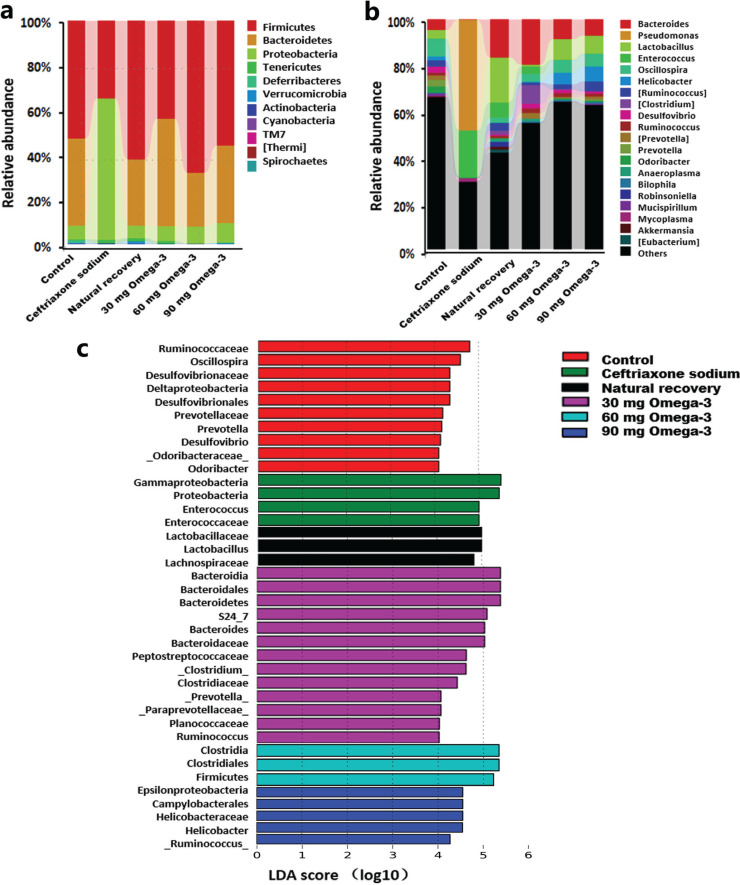
Relative abundance of cecal microbiota. Phylum (a) and genus level (b) taxonomy are presented as a percentage of total sequences. (c) The linear discriminant analysis effect size (LefSe) was adopted to identify the bacterial groups that showed significant differences in abundance among the six groups. Only taxa meeting an LDA significant threshold of >4 are shown.

To elucidate the most altered microbes due to ceftriaxone sodium and omega-3 PUFA administration, we compared the abundance of various bacterial groups at the phylum and genus levels in cecal samples. The main dominant phyla identified were Firmicutes, Bacteroidetes, Proteobacteria, Tenericutes and Actinobacteria in all the mice. The administration of ceftriaxone sodium resulted in a significant decrease of Bacteroidetes and an increase of Proteobacteria. Omega-3 PUFA administration or natural recovery could elevate the percentage of Bacteroidetes and decrease the percentage of Proteobacteria (Fig. 3a). At the genus level, there was a lower proportion of *Bacteroidales S24-7*, *Bacteroides*, *Lactobacillus*, *Lachnospiraceae*, *Ruminococcaceae*, and *Helicobacter*, and a higher proportion of *Pseudomonas* and *Enterococcus* in the ceftriaxone sodium group. Omega-3 PUFA administration or natural recovery could reverse the percentage of these microbiota compared with the ceftriaxone sodium group. In addition, the 60-mg omega-3 PUFA and the 90-mg omega-3 PUFA administration showed a better recovery effect on the microbiota than the 30-mg omega-3 PUFA administration or natural recovery because the proportion and composition of bacteria in the 60- and the 90-mg omega-3 PUFA groups were most similar to those in the control group (Fig. 3b).

To further assess the effect of omega-3 PUFA administration on the gut microbiota, the taxonomic cladogram and LDA coupled with effect size measurements (LEfSe) were applied. A total of 38 taxa showed a significant difference in their abundance among the six groups (LDA score > 4.0). As shown in Fig. 3c, compared with other groups, four microbial taxa were enriched in the ceftriaxone sodium administration group, including one bacterial phylum, one class, one family and one genus. In the natural recovery group, the abundances of three microbial taxa were increased, including two families and one genus. The administration of omega-3 PUFAs increased the abundances of 21 microbial taxa, including two bacterial phyla, three classes, three orders, seven families and six genera. Interestingly, the increased microbial taxa were different in different doses of omega-3 PUFAs administration group.

### Effect of different doses of omega-3 PUFAs administration on cecal microbiota

**Fig. 4:**
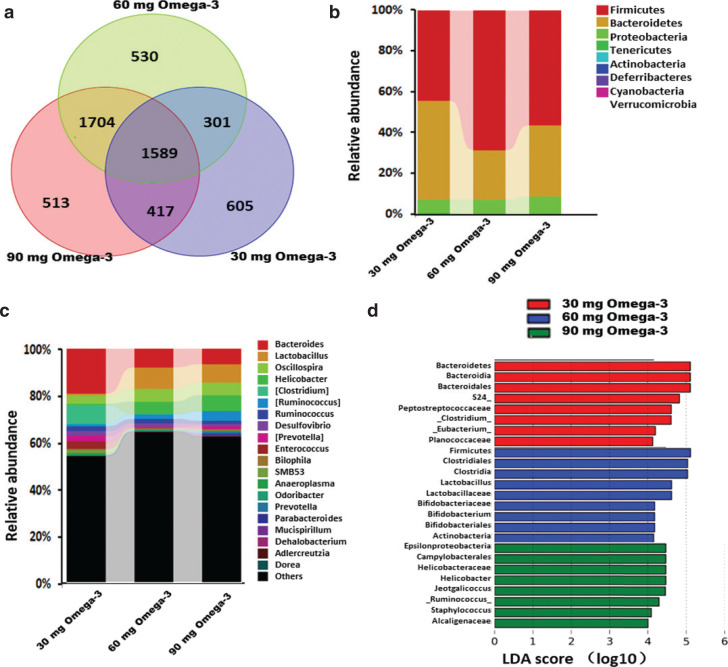
Analyses of cecal microbiota with different doses of omega-3 PUFA administration group. (a) Venn diagram of cecal microbiota in different dose omega-3 PUFAs administration group. (b) Compositions of cecal microbiota at the phylum. (c) Compositions of cecal microbiota at the genus levels. (d) LEfSe identified taxa for different doses of omega-3 PUFA administration group. Only taxa meeting an LDA significant threshold of >4 are shown.

To clarify the effects of different doses of omega-3 PUFAs on the cecal microbiota, we compared the differences in the microbiota with 30-, 60-, and 90-mg omega-3 PUFA administration. As shown in Fig. 4a, there were 605 OTUs unique in the 30-mg omega-3 PUFAs group, 530 OTUs unique in the 60-mg omega-3 PUFA group, and 513 OTUs unique in the 90-mg omega-3 PUFA group, while 1,589 OTUs were shared by the three groups. We further analyzed the abundance of different microbiota at the phylum and genus levels. The results revealed that Firmicutes were relatively more abundant, while Bacteroidetes were less abundant in the 60-mg omega-3 PUFA group when compared with those in the 30- and 90-mg omega-3 PUFA groups. At the genus level, there was a higher abundance of *Bacteroides*, *Clostridium,* and *Prevotella*, while there was a lower abundance of *Lactobacillus*, *Helicobacter,* and *Ruminococcus* in the 30-mg omega-3 PUFA group than those in the 60- and 90-mg omega-3 PUFA groups. The taxa abundance differences between the 60- and 90-mg groups were not significantly different (Fig. 4b and c).

From LEfSe analysis, it was identified that there were significantly higher abundances of *Bacteroidales.S24_7, Clostridium*, *Eubacterium,* and *Planococcaceae* in 30-mg omega-3 PUFAs group than in the 60- and 90-mg omega-3 PUFAs groups. There were significantly higher abundances of *Clostridiales*, *Lactobacillus,* and *Bifidobacterium* in the 60-mg omega-3 PUFA group than in the 30- and 90-mg omega-3 PUFA groups. However, in the 90-mg omega-3 PUFA group, there were significantly higher abundances of *Helicobacter*, *Jeotgalicoccus*, *Staphylococcus*, *Ruminococcus,* and *Alcaligenaceae* than in the 30- and 60-mg omega-3 PUFA groups (Fig. 4d).

### Omega-3 PUFA administration upregulate the expression of ZO3 and occludin in ceftriaxone sodium-exposed mice

**Fig. 5:**
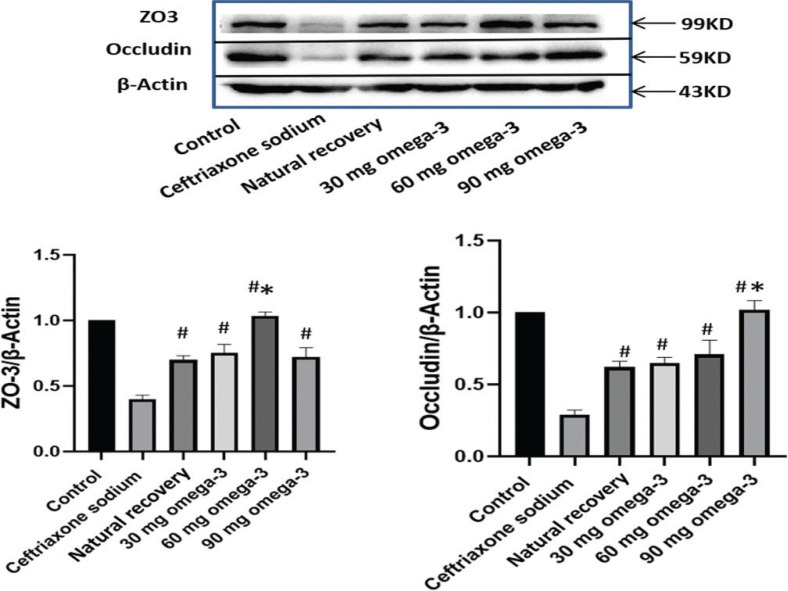
Western blot analysis of tight junction protein expression of ZO3 and occludin in mice ileal tissue. The representative bands and statistical analysis were shown. ^#^*P* < 0.05 versus ceftriaxone sodium group, *P < 0.05 versus natural recovery group.

A complete intestinal barrier function is the basis of human health. The expression level of intestinal wall tight junction proteins can affect the intercellular connection of intestinal epithelial cells, thus, affecting the permeability of intestinal wall. In this study, the expression levels of tight junction proteins, ZO3 and occludin, in the ileal tissue of mice were evaluated, and the results revealed that ceftriaxone sodium significantly downregulated the expression of ZO3 and occludin when compared with the control group. Administration of 60 mg omega-3 PUFAs could significantly upregulate the expression of ZO3 when compared with the ceftriaxone sodium-treated group and the natural recovery group. Administration of 90 mg omega-3 PUFAs could significantly upregulate the expression of occludin when compared with the ceftriaxone sodium-treated group and the natural recovery group. There was no significant difference in the expression of ZO3 and occludin in the 30-mg omega-3 PUFA administration group compared with the natural recovery group (Fig. 5).

### Omega-3 PUFA administration decreased the LPS level in ceftriaxone sodium-exposed mice

**Fig. 6:**
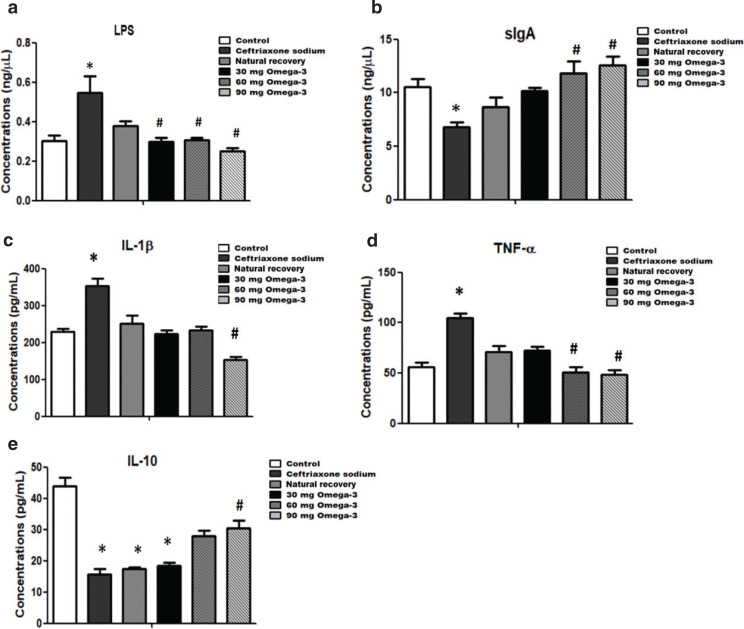
Effects of omega-3 PUFAs on the levels of serum LPS and immune parameters. (a) Serum LPS. (b) Mucus sIgA. (c) Serum IL-1β. (d) Serum TNF-α. (e) Serum IL-10. Data are presented as mean ± SEM, *P < 0.05 versus control, ^#^*P* < 0.05 versus ceftriaxone sodium group.

LPS is a component of the cell wall of gram-positive bacteria. In healthy human beings and animals, the serum LPS level is low. The serum LPS concentration can reflect the intestinal wall permeability. In this study, the serum level of LPS in the ceftriaxone sodium-treated group is significantly higher when compared with the control group. Both natural recovery and omega-3 PUFA administration could reduce the serum level of LPS, especially in the omega-3 PUFA administration groups, as the concentrations of LPS in omega-3 PUFA administration groups were significantly lower than that in the ceftriaxone sodium administration group (Fig. 6a).

### Omega-3 PUFA administration improved immune function in ceftriaxone sodium-exposed mice

Administration of ceftriaxone sodium influenced the intestinal mucosal immunity. The level of intestinal mucus SIgA was significantly lower in the ceftriaxone sodium group mice compared with the control mice. After 21 days of natural recovery or omega-3 PUFAs administration, the levels of SIgA were recovered to varying degrees, especially in 60- and 90-mg omega-3 PUFAs administration groups, as the levels of SIgA were significantly higher than that in the ceftriaxone sodium-treated group (Fig. 6b). Analysis of serum inflammatory cytokines showed that levels of IL-1β and TNF-α were significantly higher in the ceftriaxone sodium administration group compared with those in the control group. Notably, levels of these inflammatory cytokines were reduced by varying degrees in the omega-3 PUFA administration groups compared with the ceftriaxone sodium-treated group, and this effect was more significant than that in the natural recovery group, as the reduction of inflammatory cytokines was more obvious in the 60- and 90-mg omega-3 PUFAs administration groups than the natural recovery group (Fig. 6c and d). In this study, the levels of serum anti-inflammatory cytokine IL-10 were lower in the ceftriaxone sodium administration group, natural recovery group, and the 30-mg omega-3 PUFAs administration group than that in the control group. Administration of 90 mg omega-3 PUFAs significantly elevated the IL-10 level when compared with the ceftriaxone sodium-treated group (Fig. 6e).

## Discussion

In this research study, we demonstrated that omega3 PUFA administration affected the recovery of intestinal microbiota in ceftriaxone sodium-treated mice, and different doses had different effects on the microbiota. Intestinal microbiota is one of the most important components of the intestinal microecosystem (23, 24). Change in the intestinal microbiota is an important factor affecting the stability of intestinal microecosystem, as ecological theory predicts that species-rich communities are less susceptible to invasion (25). Decreasing of microbiota diversity has been correlated with some disorders, such as obesity (26), IBD (27), and acute diarrhea (28). In this study, analyses of the mice cecal microbiota α-diversity indicated that administration of ceftriaxone sodium induced a significant decrease in the gut microbiota richness. Natural recovery and 30-mg omega-3 PUFA administration had a small effect on the diversity and richness of bacteria, while the 60- and 90-mg omega-3 PUFA administration significantly increased the gut microbiota diversity and richness, which is even higher than that in the control mice. The results of β-diversity analyses also indicated that the structure of intestinal microbiota in the 60- and 90-mg omega-3 PUFA administration groups was more similar to that in the normal control group, as they clustered in a similar area. These results revealed that the regulation of omega-3 PUFAs on bacterial microbiota needs to reach a certain dose, which may be one of the reasons why a low dose of omega-3 PUFAs is ineffective in treating some diseases.

The main bacteria inhabiting the intestinal tract are belonging to four dominant phyla: Firmicutes, Bacteroidetes, Proteobacteria, and Actinobacteria. The population of these complex and dynamic microorganisms exerts a marked effect on the host during homeostasis and disease (29). Multiple factors contribute to the dynamic change of the intestinal microbiota, including geography, diet, stress, pharmaceuticals, and so on (30). Antibiotics are one of the common factors that have an obvious effect on the intestinal microbiota. Specifically, broad-spectrum antibiotics reduce bacterial diversity while expanding and declining of specific indigenous taxa (31). In this study, ceftriaxone sodium treatment reduced the abundance of Bacteroidetes and increased the abundance of Proteobacteria at the phylum level. After 21 days of natural recovery or omega-3 PUFA administration, the gut microbiota dysbiosis partially restored at the phylum level. Moreover, analysis of the data at the genus level revealed that a statistically increased abundance of *Pseudomonas*, *Enterococcus,* and *Unclassified moraxellaceae* associated with a reduction of *Unclassified_S24-7*, *Bacteroides*, *Lactobacillus*, *Unclassified_Lachnospiraceae*, *Oscillospira*, *Unclassified_Ruminococcaceae,* and *Helicobacter* in the ceftriaxone sodium administration group. Among these changing bacterial taxa, the abundance of *Pseudomonas* and *Unclassified moraxellaceae* could be completely retrieved by natural recovery and 30-mg omega-3 PUFA administration, while the recovery of other taxa requires 60- or 90-mg omega-3 PUFA administration. LEfSe analysis further identified that some microbes that play beneficial roles, such as *Lactobacillus* and *Bifidobacterium*, were increased abundance in the 60 mg omega-3 PUFAs administration group. Short-chain fatty acids (SCFAs) are important substrates for regulating the immune system and have anti-inflammatory properties. There was an increased abundance of SCFA-producing bacteria, including *Clostridia*, *Ruminococcus,* and *Helicobacter*, after 60- and 90-mg omega-3 PUFA administration. These data indicated that different doses of omega-3 PUFAs seem to work on different microbes. In 60- and 90-mg omega-3 PUFA administration groups, the generally thought to be beneficial microbes increased the abundance, which may be the basis for the beneficial effects of omega-3 PUFAs.

A healthy gut microbiome plays a major role in the overall health of the host. Changes in the gut microbiota are often accompanied by changes in gut permeability (32, 33). Intestinal toxic digestive metabolites, bacterial toxins, and small molecules enter the bloodstream through the leaky gut and adversely affect the host immune system (34). Tight junction proteins, such as cytoplasmic scaffolding proteins ZO family and occludins, play crucial roles in maintaining the gut barrier integrity and function. In this study, administration of ceftriaxone sodium increased the gut permeability as the expression of the tight junction protein ZO3 and occludin significantly decreased and gram-positive bacteria metabolites LPS concentration increased in the blood. Administration of 60 and 90 mg omega-3 PUFAs could increase the expression of ZO3 and occludin, respectively. Serum levels of LPS reduced in 60- and 90-mg omega-3 PUFAs groups, suggesting that administration of omega-3 PUFAs could reinforce the intestinal barrier. Administration of 30 mg omega-3 PUFAs had no significant effect on the expression of tight junction proteins, indicating that a certain amount of omega-3 PUFAs was needed to play an effective role.

Gut microbiota is essential for the modulation of the immune system. Changes in the intestinal microbiota are linked to the inflammation state (35). In this study, administration of 60- and 90-mg omega-3 PUFAs could induce the secretion of the intestinal mucosa immunoglobulin sIgA, which could, in turn, enhance the body’s intestinal mucosal immunity. IL-1β and TNF-α were considered to be the most potent pro-inflammatory cytokines that trigger inflammation in diseases (36, 37). A supplement of 90 mg omega-3 PUFAs could decrease the concentrations of IL-1β and TNF-α in the serum. IL-10 is an essential anti-inflammatory cytokine that negatively regulates the immune response to antigens. The deficiency of IL-10 is associated with a severe inflammatory state of the gut (38, 39). In this study, only a 90-mg omega-3 PUFA supplement could significantly increase the IL-10 concentration. These data suggested that a certain concentration of omega-3 PUFAs is needed to regulate immunity.

The limitation of this study is that the omega-3 status was not measured. The administration of omega-3 PUFAs was through gavage. Oral bioavailability of the fatty acids was low due to the low water solubility. Nevertheless, the conventional way of fatty acid intake is through the digestive tract. Intragastric administration can best mimic the human intake process; meanwhile, intragastric administration can determine the exact dose of fatty acids entering the body.

## Conclusions

This study showed that supplementation with omega-3 PUFAs was beneficial to restore the gut microbiota in ceftriaxone sodium-treated mice. The 60- and 90-mg omega-3 PUFAs supplementation groups have a better recovery effect than the 30-mg group. Moreover, the 60- and 90-mg omega-3 PUFA supplementation could regulate the immunity. This research study hints that different doses of omega-3 PUFAs have different effects on bacteria and immunity; high-dose omega-3 PUFAs could play a beneficial role in the body. Further explorations for understanding the optimal dose of omega-3 PUFA supplementation are still needed.
